# miR-17-5p suppresses cell proliferation and invasion by targeting ETV1 in triple-negative breast cancer

**DOI:** 10.1186/s12885-017-3674-x

**Published:** 2017-11-10

**Authors:** Jie Li, Yuanhui Lai, Jieyi Ma, Yue Liu, Jiong Bi, Longjuan Zhang, Lianzhou Chen, Chen Yao, Weiming Lv, Guangqi Chang, Shenming Wang, Mao Ouyang, Wenjian Wang

**Affiliations:** 1 Laboratory of Department of Surgery, First Affiliated Hospital of Sun Yat-sen University, 58 Zhongshan Rd II, Guangzhou, Guangdong 510080 People’s Republic of China; 2 Department of Vascular, Thyroid and Breast Surgery, First Affiliated Hospital of Sun Yat-sen University, 58 Zhongshan Rd II, Guangzhou, Guangdong 510080 People’s Republic of China; 3 Department of Vascular, Thyroid and Breast Surgery, Eastern Hospital of the First Affiliated Hospital of Sun Yat-sen University, 183 East Huangpu Road, Guangzhou, Guangdong 510080 People’s Republic of China; 4 Centre for Cellular & Structural biology, School of Pharmaceutical Sciences of Sun Yat-Sen University, 132 East Waihuan Road, Guangzhou, Guangdong People’s Republic of China; 5 Department of Clinical Laboratory, First Affiliated Hospital of Sun Yat-sen University, 58 Zhongshan Rd II, Guangzhou, Guangdong 510080 People’s Republic of China

**Keywords:** miR-17-5p, ETV1, Triple-negative breast cancer

## Abstract

**Background:**

Triple-negative breast cancer (TNBC) is the malignancy with the worst outcome among all breast cancer subtypes. We reported that ETV1 is a significant oncogene in TNBC tumourigenesis. Consequently, investigating the critical regulatory microRNAs (miRNAs) of ETV1 may be beneficial for TNBC targeted therapy.

**Methods:**

We performed in situ hybridization (ISH) and immunohistochemistry (IHC) to detect the location of miR-17-5p and ETV1 in TNBC patient samples, respectively. miR-17-5p expression in TNBC tissues and cell lines was assessed by quantitative real-time PCR (qRT-PCR). ETV1 expression was evaluated by qRT-PCR, western blotting and IHC. Cell Counting Kit-8 (CCK-8), colony formation, Transwell and wound closure assays were utilized to determine the TNBC cell proliferation and migration capabilities. In vivo tumour metastatic assays were performed in a zebra fish model.

**Results:**

The abundance of miR-17-5p was significantly decreased in TNBC cell lines and clinical TNBC tissues. The miR-17-5p expression levels were closely correlated with tumour size (*P* < 0.05) and TNM stage (*P* < 0.05). By contrast, the expression of ETV1 was significantly up-regulated in TNBC cell lines and tissues. There is an inverse correlation between the expression status of miR-17-5p and ETV1 (*r* = −0.28, *P* = 3.88 × 10^−3^). Luciferase reporter assay confirmed that ETV1 was a direct target of miR-17-5p. Forced expression of miR-17-5p in MDA-MB-231 or BT549 cells significantly decreased ETV1 expression and suppressed cell proliferation, migration in vitro and tumour metastasis in vivo. However, rescuing the expression of ETV1 in the presence of miR-17-5p significantly recovered the cell phenotype. High miR-17-5p expression was associated with a significantly favourable prognosis, in either the ETV1-positive or ETV1-negative groups (log-rank test, *P* < 0.001; *P* < 0.001). Both univariate and multivariate analyses showed that miR-17-5p and ETV1 were independent risk factors in the prognosis of TNBC patient.

**Conclusions:**

Our data indicate that miR-17-5p acts as a tumour suppressor in TNBC by targeting ETV1, and a low-abundance of miR-17-5p may be involved in the pathogenesis of TNBC. These findings indicate that miR-17-5p may be a therapeutic target for TNBC.

**Electronic supplementary material:**

The online version of this article (10.1186/s12885-017-3674-x) contains supplementary material, which is available to authorized users.

## Background

Triple-negative breast cancer (TNBC) is a challenging disease with the worst outcome among all breast cancer subtypes worldwide [1]. It has the highest rate of relapse within 1–3 years despite adjuvant chemotherapy [2]. Accordingly, identifying and assessing additional critical factors that may affect the outcome of TNBC therapy is worth continuous efforts.

ETV1 (ETS variant 1), one member of PEA3 subfamily of ETS transcription factors, is a well-known oncogene for a variety of human malignant diseases [3–5]. We reported that ETV1 promotes triple-negative breast tumour cell proliferation, invasion and migration and is an independent, poor prognostic predictor of TNBC patients [6]. Therefore, the factors that regulate ETV1 expression were investigated as a potential approach for TNBC targeted therapy.

MicroRNAs (miRNAs) are firmly established as master regulators of the human genome [7]. Their aberrant expression is crucial for cancer initiation, progression and dissemination by controlling expression of their targets [8]. The expression status of miRNAs is likely derived from their widely different gene expression profiles in each tissue. They may function as oncogenes or tumour suppressor genes in a tissue-dependent matter [9]. Based on Targetscan prediction, we found that ETV1 transcription may be controlled by miR-17-5p [10]. miR-17-5p belongs to the miR-17-92 cluster, which plays a critical role in tumourigenesis [11]. It has been confirmed that miR-17-5p can function as an oncogene or tumour suppressor by targeting ETV1 in melanoma or GIST [9, 12]. Whether miR-17-5p contributes to triple-negative breast tumour cell function via ETV1 targeting has not yet been reported.

In this study, we investigated the expression status of both miR-17-5p and ETV1 and their association in TNBC. The abundance of miR-17-5p is significantly decreased in TNBC, which is in contrast to ETV1 expression. We demonstrated that ETV1 is a direct target of miR-17-5p. Furthermore, up-regulation of miR-17-5p suppresses TNBC cell growth, migration and invasion in vitro, and metastasis in vivo by targeting ETV1. miR-17-5p enhancement is a independent favourable prognostic factor for TNBC patients in contrast to ETV1. This provides a new opportunity to improve TNBC patient treatment efficiency.

## Methods

### Patients and specimens

Paired TNBC and adjacent non-tumour tissues were collected from 105 patients who underwent surgical resection at the First Affiliated Hospital of Sun Yat-sen University from January 1997 to December 2007. No patient received preoperative radiotherapy or chemotherapy. Tumor stages and clinicopathological classifications were defined with the pathologic tumor-node-metastasis (pTNM) classification [6]. All specimens were fixed with paraformaldehyde or snap-frozen in liquid nitrogen.

### Cell culture, lentivirus and oligonucleotides transfection

Breast cancer cell lines, MCF 10A (CRL-10317™), MDA-MB-231 (HTB-26™), BT-549 (HTB-122™), Hs 578 T (HTB-126™), were purchased from the American Type Culture Collection (ATCC, Manassas, USA) and cultured in DMEM (Gibco, USA) supplemented with 10% foetal bovine serum (Gibco, Germany), 1% penicillin and 1% streptomycin (Invitrogen, USA). Cells were maintained at 37°C in a humidified atmosphere containing 5% carbon dioxide. The human ETV1 coding sequence was cloned into the GV141 vector (GV141-ETV1). miR-17-5p was cloned into the GV248 vector containing GFP and was packaged into a lentivirus by Genechem (Genechem Co., China) (LV-miR-17-5p). The primers are displayed in Additional file 1: Table S1. Lentivirus with the empty vector was used as a negative control (LV-NC). The viruses were transfected into cells according to the manufacturer’s instructions. The miR-17-5p mimic, inhibitor and negative control oligos were purchased from RiboBio (China) and transfected into cells using Lipofectamine RNAiMAX (Invitrogen, USA) according to the manufacturer’s instructions. The transfection efficiency of the cells was determined using qRT-PCR.

### Quantitative real time PCR (qRT-PCR) analysis

qRT-PCR analyses of miR-17-5p and ETV1 expression were performed on a LightCycler 480 (Roche Diagnostics, Germany) according to our previous report [13]. The relative expression of miR-17-5p in 105 pairs of TNBC/adjacent non-tumour tissues was divided into miR-17-5p-low expression group and -high expression group according to the relative ratio of miR-17-5p expression in tumour/adjacent non-tumour tissues < or > 0.5. All reactions were run in triplicate. The level of miR-17-5p was normalized to U6, and the level of ETV1 was normalized to GAPDH. Primer sequences used in the assays are listed in Additional file 2: Table S2.

### In situ hybridization and immunohistochemical staining

The localization of miR-17-5p and ETV1 was observed by in situ hybridization (ISH) and immunohistochemistry (IHC), respectively. In situ observation of miR-17-5p was performed using 4-μm sections of samples with a digoxigenin-labelled oligonucleotide miR-17-5p detection probe (Exiqon, USA), as previously described [13]. miR-17-5p probe sequence was (5′-3′) CTACCTGCACTGTAAGCACTTTG. Immunohistochemical staining of ETV1 was performed according to the manufacturer’s protocol on paired TNBC and adjacent normal tissues. The negative control was performed by replacing the primary antibody with preimmune rabbit serum. The immunoreactivity score method based on the proportion and intensity of positively stained tumor cells was employed according to our previous description [6].

The following antibodies were used for immunohistochemical staining and western blotting in this study: anti-GAPDH (ab181602)) and anti-ETV1 antibodies (ab81086) were purchased from Abcam (USA). Appropriate secondary antibodies were obtained from Cell Signaling Technology (USA).

### Western blotting analysis

To investigate the effect of miR-17-5p on ETV1 protein expression, western blotting was conducted. The cell samples were lysed in RIPA protein lysis buffer (Beyotime, China) supplemented with 1 mM PMSF. The protein concentration was determined by NanoDrop at 280 nm (Thermo, USA). Equal amounts of protein (25 μg) were loaded and separated on 10% SDS polyacrylamide gels under denaturing conditions and transferred onto a PVDF membrane (Millipore, USA). After blocking with 5% non-fat milk in TBS, the membrane was incubated with ETV1 primary antibody overnight at 4°C. Then, the membrane was washed and incubated with secondary horseradish peroxidase (HRP)-conjugated antibody for 1 h at room temperature. GAPDH was used as an internal control. The immunoreaction was visualized by ECL (Millipore, USA) and imaged using a GE ImageQuant Las 4000 mini (GE, USA).

### Luciferase assay

Fragments of the ETV1 mRNA 3′-UTRs containing the putative or mutated miRNA binding sites for miR-17-5p were cloned into the GV306 luciferase reporter vector (GeneChem, China). The constructs were then co-transfected with miR-17-5p mimic or negative control oligos into 293 T cells using Lipofectamine 2000 (Invitrogen, USA) according to the manufacturer’s instructions. Luciferase activity was measured 48 h after transfection using the Dual-Luciferase® Reporter Assay System(Promega, USA)according to the manufacturer’s protocol.

### Cell colony formation and proliferation assays

We utilized the colony formation and Cell Counting Kit-8 (CCK-8, Sigma) assays to determine the TNBC cell proliferative ability. MDA-MB-231 and BT549 cells were infected with 1 × 10^8^ TU/ml LV-miR-17-5p or LV-NC in enhanced infection solution containing 5 μg/μl polybrene (Genechem Co., China). For the colony formation assay, the transfected cells (300/well) were allowed to grow in 6-well plates and maintained in medium containing 10% FBS, and the medium was replaced every 2 days. After 14 days, cells were fixed with 4% formaldehyde and stained with 0.1% crystal violet at room temperature for 30 min. Colonies were counted only if they included at least 50 cells, and they were then compared.

For the CCK8 assay, the transfected cells (3 × 10^3^ cells/well) were seeded in 96-well plates and measured every 24 h following the manufacturer’s instruction. Absorbance was read at 450 nm [6].

For the restoration of ETV1 expression, GV141-ETV1 was transfected into the cells in the presence of miR-17-5p with Lipofectamine RNAiMAX (Invitrogen, USA) according to the manufacturer’s protocol.

All experiments were performed in triplicate.

### Cell migration and invasion ability analyses

The migration and invasion abilities of TNBC cells were observed using Transwell and wound healing assays. Transwell assays were performed in cell culture inserts with a PET membrane (Corning, USA). At 48 h after virus transfection, the cells (1 × 10^5^ cells) were resuspended in serum free medium and added to the upper chamber of an insert. Then, the insert was placed in a 24-well plate containing DMEM supplemented with 10% foetal bovine serum. Cells were incubated for 24 h for the migration assay and 48 h for the invasion assay. After the cells were incubated for the exact time at 37 °C, the inserts were washed with PBS, and cells on the upper surface of the insert were removed with a cotton swab. Cells adhering to the lower surface were fixed with 4% formaldehyde for 30 min, stained with 0.1% crystal violet solution and imaged under a microscope (ZEISS Axio Observer Z1, Jena, Germany).

For the wound healing assay, the virus transfected cells (1 × 10^5^ cells) were seeded into a 6-well plate. Upon reaching 100% confluence, the cell layer was scratched with a 200 μl pipette tip and washed with culture medium twice and cultured again for up to 48 h. Finally, the scratch wounds were visualized and imaged under a microscope (ZEISS Axio Observer Z1, Jena, Germany).

All experiments were performed at least three times.

### Zebrafish metastatic model

The effect of miR-17-5p on invasive and metastatic features of TNBC cells was evaluated using a zebrafish metastatic model [14, 15]. The study was approved by the Animal Care and Use Committee of Sun Yat-sen University. Briefly, negative control oligos or miR-17-5p mimic transfected MDA-MB-231 cells were labelled with the fluorescent dye CM-Dil (Life Technologies, USA). At 48 h-post fertilisation (hpf), zebrafish embryos were anaesthetized with 0.003% tricaine (Sigma, USA), and approximately 200 transfected cells were microinjected at approximately 60 μm above the ventral end of the duct of Cuvier, perivitelline spaces, using a pressure microinjector. The embryos were incubated at 33 °C and then anaesthetized and imaged using microscopy at 48 h after injection.

### Statistics

Data are expressed as the means ±SEM. The R 3.1.2 software package was used for all the statistical calculations. The Fisher’s exact test was used to analyse the relationship between miR-17-5p and ETV1 expression and clinicopathological features. The differences between groups were analysed using Student’s t test when there were only two groups, or were assessed by one-way ANOVA when there were more than two groups. Survival curves were evaluated using the Kaplan-Meier method. Cox proportional hazard regression models were used to evaluate the association of miR-17-5p and ETV1 status with survival outcomes after adjusting for covariates. Univariate and multivariate Cox regression analyses were used to find independent prognostic factors. The results with *P* values <0.05 were considered statistically significant.

## Results

### Expression status of miR-17-5p is inversely related to ETV1 and is proportional to the prognoses of TNBC patients

**Fig. 1:**
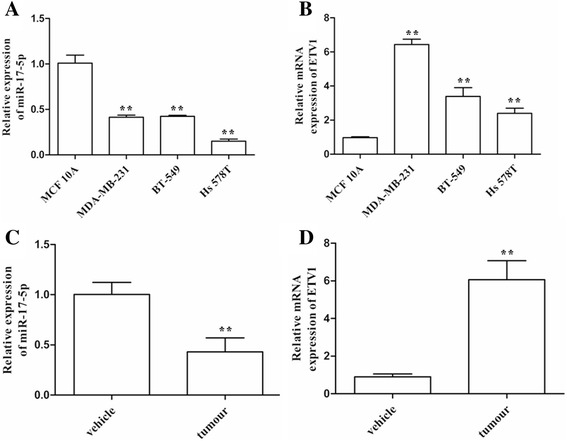
Features of miR-17-5p and ETV1 expression in TNBC cells and clinical samples. **a**, **b**, QRT-PCR analyses of miR-17-5p and ETV1 expression levels in TNBC cells. **c**, **d**, The expression levels of miR-17-5p and ETV1 in clinical TNBC samples evaluated by qRT-PCR. Data are expressed as the mean ± SEM of three independent experiments. **P < 0.01, compared with the control

**Fig. 2:**
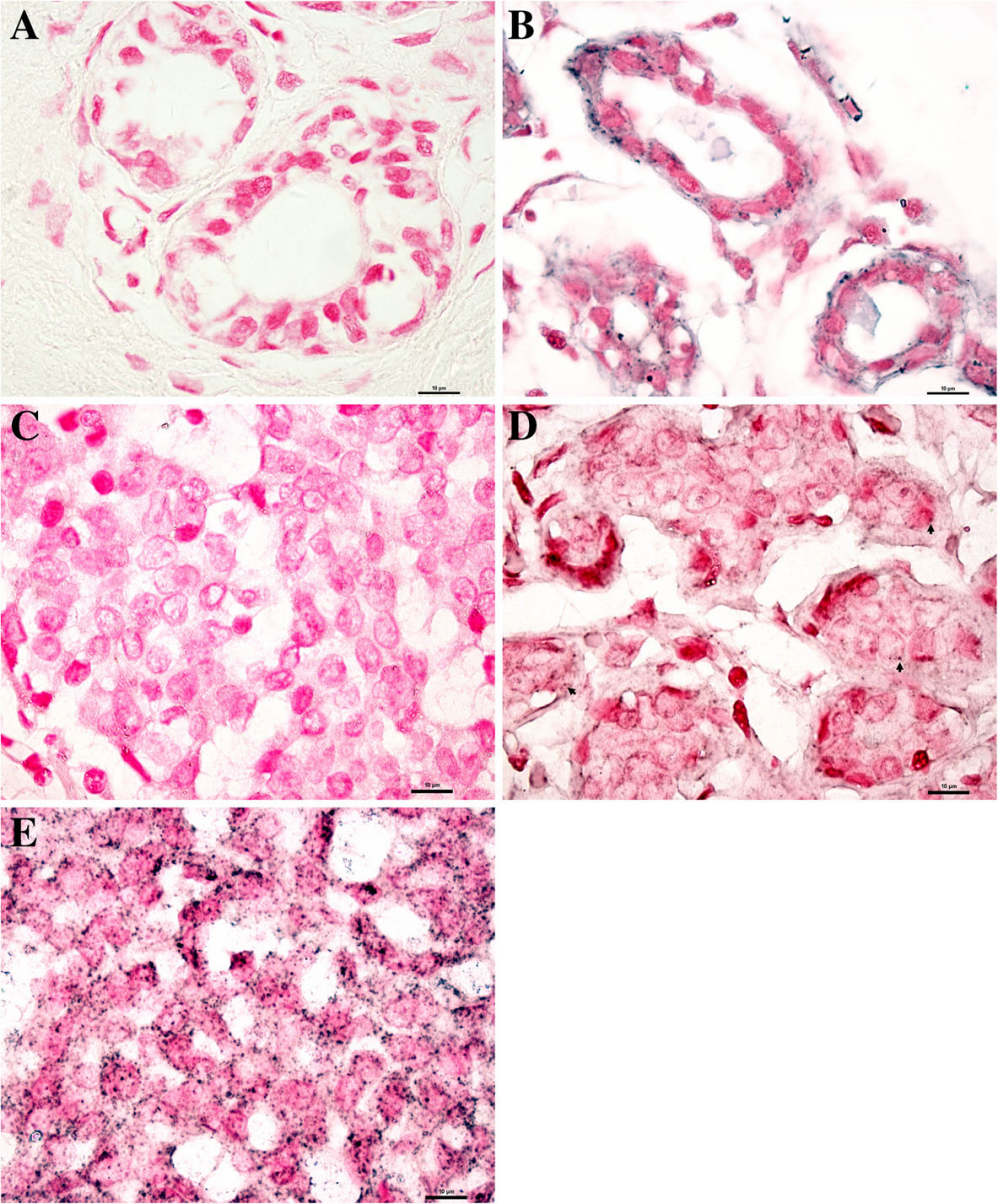
The expression status of miR-17-5p in clinical TNBC samples detected by In situ hybridization. **a**, **c**, Negative control of miR-17-5p in TNBC and adjacent non-tumour tissues. Positive expression of miR-17-5p in TNBC (**d, e**) and non-tumour tissues (**b**) was also presented. Scale bar, 10 μm

**Fig. 3:**
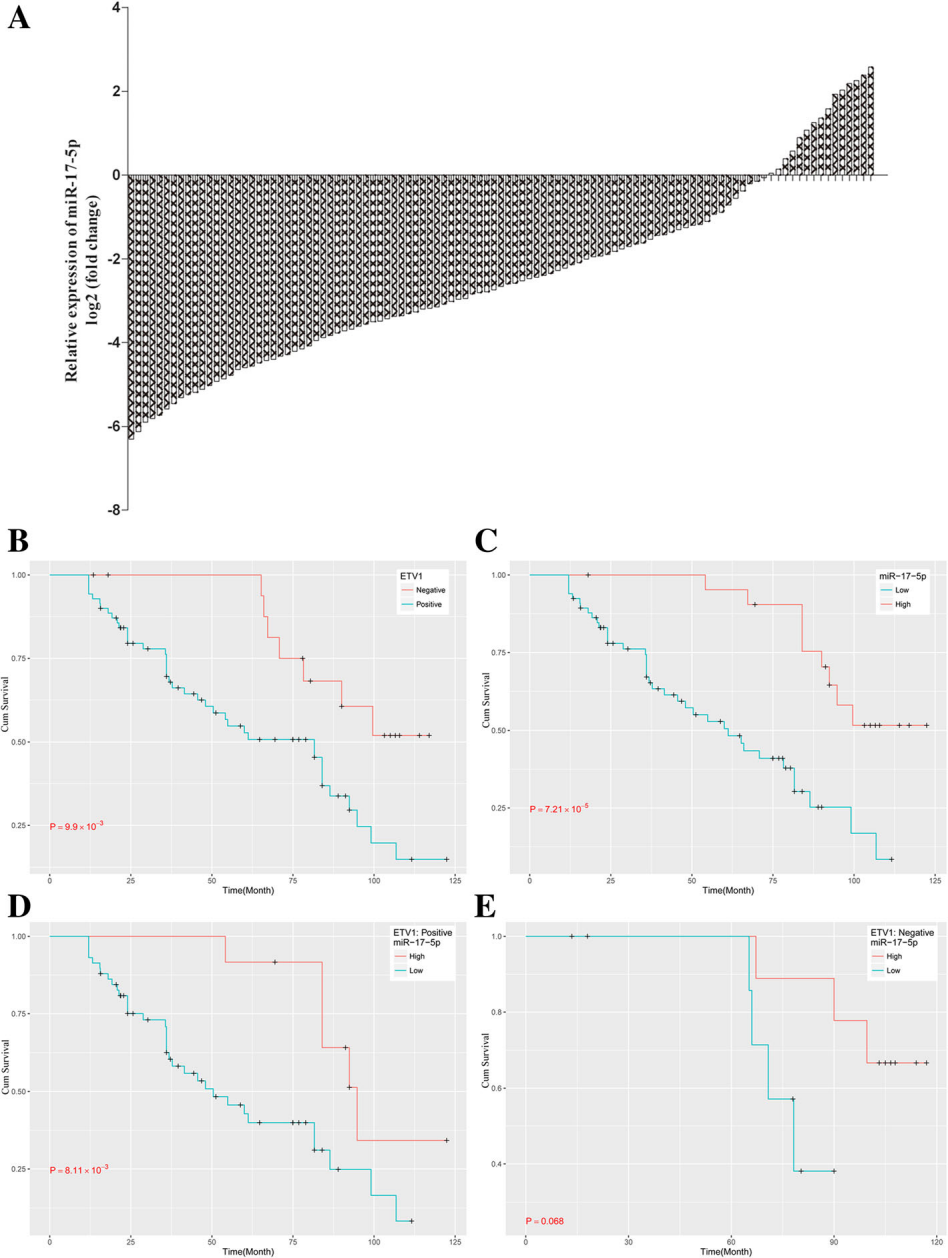
Overall survival (OS) of TNBC patients according to miR-17-5p and ETV1 expression status. **a**, The relative expression of miR-17-5p in 105 pairs of TNBC/adjacent non-tumour tissues was significantly lower. **b**, Significant difference in OS was observed between ETV1-positive (*n* = 79) and -negative (*n* = 26) expression group (*P* < 0.001). **c**, Significant difference in OS was observed between miR-17-5p-high (*n* = 23) and -low (*n* = 82) expression group (*P* < 0.001). **d**, In 79 cases of ETV1-positive TNBCs, miR-17-5p-high cases had significant higher survival rate than those of miR-17-5p-low group (*P* < 0.001). **e**, In 26 cases of ETV1-negative TNBCs, miR-17-5p-high cases had significant higher survival rate than those of miR-17-5p-low group (*P* < 0.001)

miR-17-5p expression patterns vary with tumour types [12, 16, 17]. To investigate the correlation between the expression of miR-17-5p and ETV1 in TNBC, we evaluated the abundance of miR-17-5p by qRT-PCR in TNBC cell lines and 105 TNBC patients’ samples. The expression of ETV1 was tested by qRT-PCR, western blotting and IHC with equivalent samples. The expression levels of miR-17-5p were significantly decreased in TNBC cell lines and tumour tissues [Fig. 1A, C, and Fig. 3A]. This was further confirmed by ISH [Fig. 2]. The ETV1 mRNA and protein levels were significantly increased in TNBC cell lines and tissues [Fig. 1B, D and Additional file 3: Fig. S1], which was inversely correlated with miR-17-5p (*r* = −0.28, *P* = 3.88 × 10^−3^) [Table 1].

**Table 1:** The expression of ETV1 and miR-17-5p in the tissue of TNBC

	miR-17-5p			
ETV1	High	Low	OR	*P* ^*^
Negative	10	16		
Positive	13	66	3.13	0.028

*P* ^*^: Fisher’s Exact Test

As shown in Table 2, the expression levels of miR-17-5p were significantly correlated with tumour size (*P* < 0.05) and TNM stage (*P* < 0.05). ETV1 was closely related to TNM stage (*P* < 0.05), lymph nodes involved (*P* < 0.05) and tumour relapse (*P* < 0.01) [Additional file 4: Table S3]. Kaplan–Meier curves and log-rank test showed that women in the miR-17-5p-low expression group (*n* = 82) had a shorter overall survival (OS) time than those in the miR-17-5p-high expression group (*n* = 23) (log-rank test, *P* < 0.001), which was consistent with ETV1-positive tumour group [Fig. 3B, C]. In 79 ETV1-positive tumours, miR-17-5p-high expression cases had significantly higher survival rates than patients with miR-17-5p-low expression (log-rank test, *P* < 0.001) [Fig. 3D]. Women with ETV1-negative/miR-17-5p-high tumours had the best survival relative to women with other subtypes (log-rank test, *P* < 0.001) [Fig. 3E]. The results indicate that miR-17-5p is a favourable prognostic factor, while ETV1 is a poor prognostic factor for the patient. To validate whether the expression statuses of miR-17-5p or ETV1 are independent prognostic predictors of OS for TNBC patients, we performed univariate and multivariate Cox regression analyses respectively. In univariate Cox regression analysis, the Cox proportional hazards model was adjusted for age, tumour size, pathological type, number of lymph node metastasis and TNM stage. The results showed that the miR-17-5p and ETV1 expression statuses, and patients’ age and TNM stage were significantly associated with patients OS [Table 3]. Furthermore, multivariate Cox regression analysis, adjusted for age and TNM stage, also confirmed that miR-17-5p and ETV1 expression statuses, and TNM stage were significantly associated with patients OS [Table 4]. These data suggest that miR-17-5p and ETV1 are independent prognostic predictors of OS for TNBC patient.

**Table 2:** Correlation between miR-17-5p expression and clinicopathologic features of TNBC patients

Characteristics	*n*	miR-17-5p		*P* value
		High	Low	
Age (y)				
≤ 50	63	14 (22%)	49 (78%)	1.000
> 50	42	9 (21%)	33 (79%)	
Tumour Size				
≤ 2 cm	19	8 (42%)	11 (58%)	0.030^*^
> 2 cm	86	15 (17%)	71 (83%)	
Pathological type				
Invasive ductal carcinomas	93	19 (20%)	74 (80%)	0.292
Others	12	4 (33%)	8 (67%)	
TNM Stage				
I	6	3 (50%)	3 (50%)	0.022^*^
II	34	11 (32%)	23 (68%)	
III	65	9 (14%)	56 (86%)	
Lymph node metastasis				
0–4	45	15 (33%)	30 (67%)	0.050
5–8	39	6 (15%)	33 (85%)	
≥ 9	21	2 (10%)	19 (90%)	
Relapse				
No	67	18 (27%)	49 (73%)	0.141
Yes	38	5 (13%)	33 (87%)	

*means *P* < 0.05; Fisher exact test

**Table 3:** Results of univariate analysis of over survival (OS) for patients with TNBC

Characteristics	HR	95% CI	*P* value
miR-17-5p	0.08	0.02–0.31	2.32 × 10^−4^
ETV1	4.10	1.07–15.68	0.040
Age (y)			
≤ 50 (ref)			
> 50	1.95	1.09–3.49	0.025
Tumour Size			
≤ 2 cm (ref)			
> 2 cm	1.57	0.70–1.92	0.276
Pathological Type			
IDC (ref)			
Others	0.68	0.24–1.92	0.472
Lymph node metastasis			
0–4 (ref)			
5–8	1.68	0.89–3.17	0.108
≥ 9	1.44	0.62–3.33	0.391
TNM			
I + II (ref)			
III	3.52	1.69–7.34	7.60 × 10^−4^

**Table 4:** Results of multivariate analysis of over survival (OS) for patients with TNBC

Characteristics	HR	95% CI	*P* value
miR-17-5p			
High (ref)			
Low	2.56	1.14–5.76	0.023
ETV1			
Negative (ref)			
Positive	2.12	0.88–5.08	0.093
Age (y)			
≤ 50 (ref)			
> 50	1.78	0.94–3.38	0.076
TNM			
I + II (ref)			
III	1.82	0.80–4.13	0.151

The results suggest that the expression pattern of miR-17-5p is inversely correlated with that of ETV1 in TNBC, which there might exist interaction between miR-17-5p and ETV1. miR-17-5p is a independent favourable predictor for the prognoses of TNBC patients in contrast to ETV1.

### ETV1 is a direct target of miR-17-5p

**Fig. 4:**
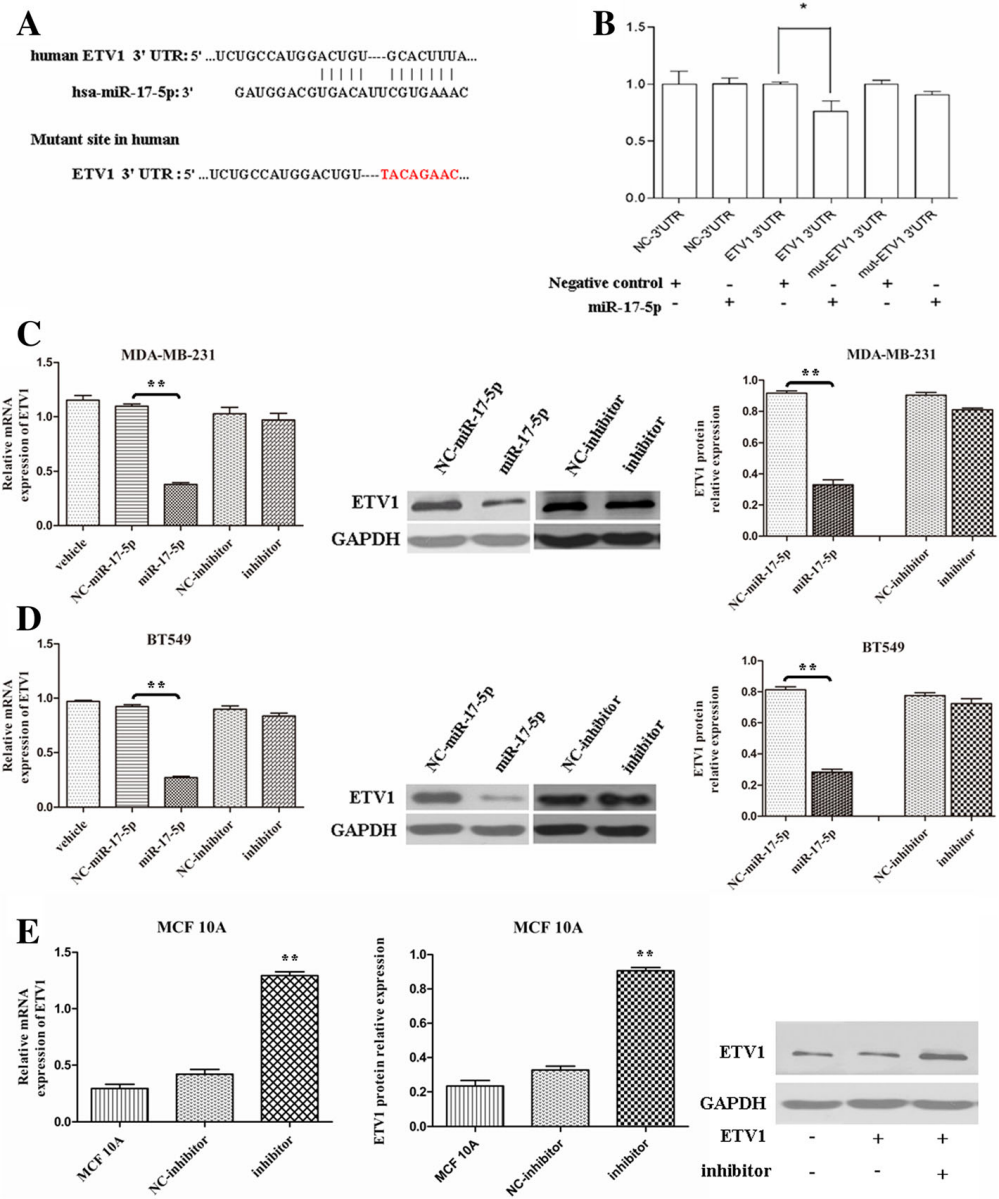
ETV1 is a direct target of miR-17-5p in TNBC cells. **a**, Target sequences of miR-17-5p in ETV1 3′-UTR and mutant sites in 3′-UTR. **b**, Relative luciferase activity of ETV1 3′-UTR and mutant in the miR-17-5p mimic-transfected 293 T cells. **c**, **d**, The effect of miR-17-5p on ETV1 expression in MDA-MB-231 and BT549 cells was detected by qRT-PCR and western blotting after the cells were transfected with miR-17-5p mimic or inhibitor, respectively. **e**, The effect of miR-17-5p on ETV1 expression was also observed in MCF10A cells by co-transfecting with GV141-ETV1 and miR-17-5p inhibitor. Data are expressed as the mean ± SEM of three independent experiments. *P < 0.05, **P < 0.01

ETV1 is a significant oncogene in TNBC as we previously reported [6]. Accordingly, identifying its critical regulators may be a promising approach for TNBC targeted therapy. To address this, we first utilized the Targetscan system and predicted that ETV1 is a potential target of miR-17-5p, which exhibits seed sequence complementary to miR-17-5p [Fig. 4A]. This is consistent with the literature [8, 11]. To confirm whether ETV1 is a direct target of miR-17-5p, we performed a luciferase reporter assay. The luciferase activity was significantly reduced by miR-17-5p in the presence of the ETV1 3’UTR (wild-type) compared to the negative control. Moreover, the inhibitory effect of miR-17-5p on the luciferase activity was abrogated when we mutated the miR-17-5p binding site in the 3’UTR of ETV1 mRNA [Fig. 4B]. These data suggest that ETV1 is a direct target of miR-17-5p.

miRNAs regulate the target genes by either degrading their mRNAs or inhibiting their proteins translation [18]. miR-17-5p degrades ETV1 expression at the protein level in melanoma cells, and at the mRNA level in GIST cells [9, 12]. We confirmed that miR-17-5p is significantly down-regulated in TNBC cells, whereas ETV1 is significantly up-regulated [Fig. 1]. To investigate the modulatory mode of miR-17-5p on ETV1 in TNBC cells, we treated MDA-MB-231 and BT549 cells with miR-17-5p mimic (50 nmol/L) or inhibitor (100 nmol/L), respectively. In addition, we co-transfected MCF10A cells with GV141-ETV1 (15 μg/μl) and miR-17-5p inhibitor (100 nmol/L). As shown in Fig. 4C-E, miR-17-5p significantly decreased both the ETV1 mRNA and protein levels, consistent with the report [12]. However, the miR-17-5p inhibitor did not affect the ETV1 mRNA and protein levels in TNBC cells [Fig. 4C, D]. We speculate that it may be because of the lower level of miR-17-5p in TNBC cells, which would not respond to further inhibition of miR-17-5p. This was further validated in MCF10A cells [Fig. 4E].

### miR-17-5p inhibits TNBC cells proliferation, migration and invasion

**Fig. 5:**
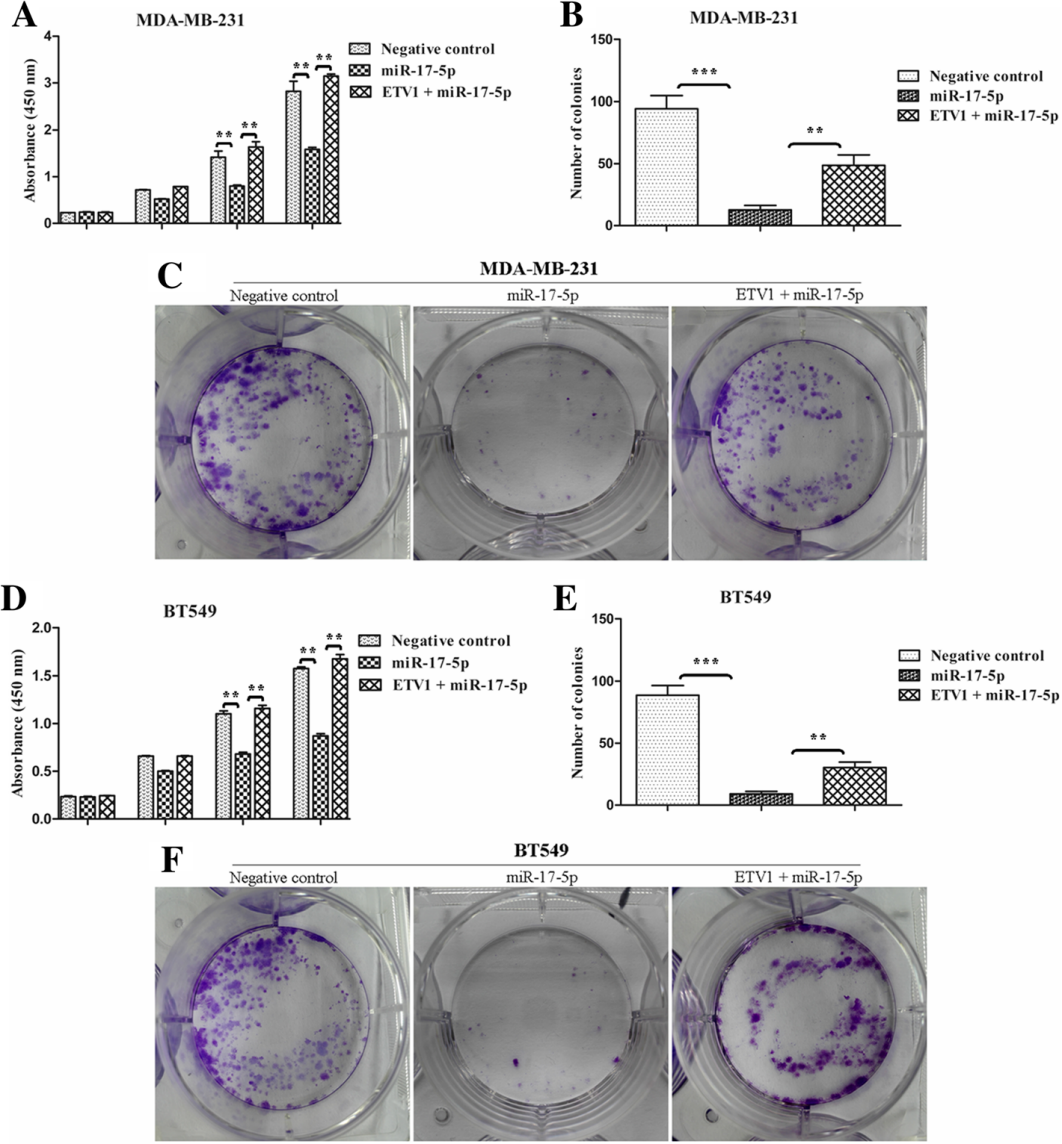
miR-17-5p suppressed TNBC cells proliferation by targeting ETV1 in vitro. MDA-MB-231 and BT549 cells were transfected with with LV-miR-17-5p or LV-NC. Colony formation and CCK8 assays were used to observe the proliferative ability changes of the cells. **b, c, e, f,** miR-17-5p led to a significant reduction of colony numbers in TNBC cells. Restoration of ETV1 expression in the presence of miR-17-5p significantly rescued the colony potential of the cells. **a**, **d**, Similar outcomes derived from the CCK8 assays. Data are expressed as the mean ± SEM of three independent experiments. **P < 0.01, ***P < 0.001

**Fig. 6:**
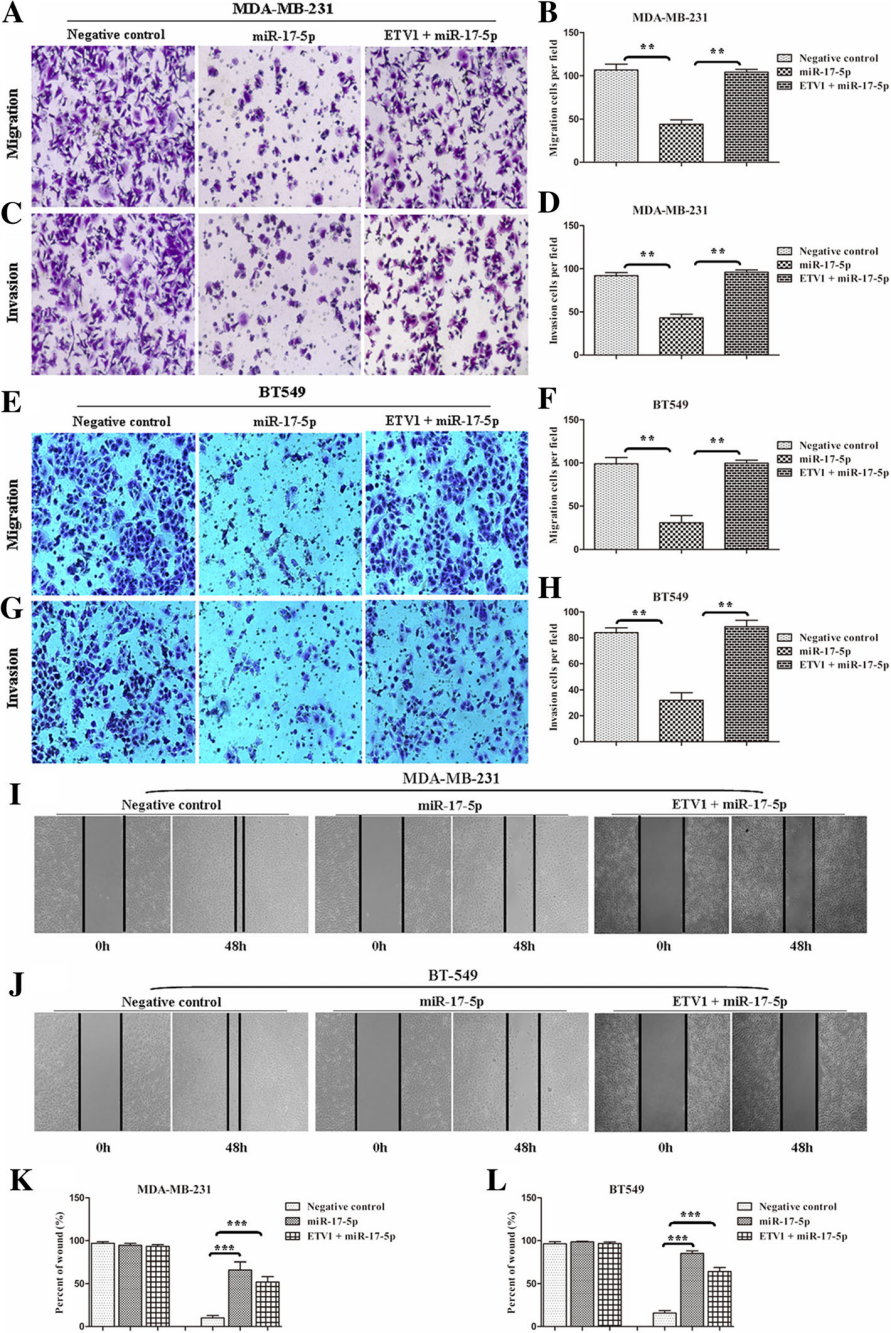
miR-17-5p inhibited TNBC cells migration and invasion capacities by targeting ETV1 in vitro. MDA-MB-231 and BT549 cells were transfected with LV-miR-17-5p or LV-NC. miR-17-5p decreased the number of migrating and invasive MDA-MB-231 and BT549 cells. Restoration of ETV1 expression in the presence of miR-17-5p significantly recovered the cells migratory and invasive capabilities observed with Transwell (**a**-**h**) and wound closure (**i**-**l**) assays. Data are expressed as the mean ± SEM of three independent experiments. **P < 0.01, ***P < 0.001. Original magnification: 100 ×

To examine the biological effects of miR-17-5p on TNBC cells and to achieve a higher abundance of miR-17-5p, we transfected MDA-MB-231 and BT549 cells with LV-miR-17-5p or LV-NC. Then, we utilized colony formation and CCK8 assays to observe the proliferative ability changes of the cells, and utilized Transwell and wound closure assays to check the changes of the cells migratory capacities. Consequently, forced miR-17-5p expression led to a significant reduction in the number of colonies for the TNBC cells [Fig. 5B, C, E, F]. Consistent with the colony formation assays, up-regulation of miR-17-5p significantly suppressed the cells proliferation according to the CCK8 analyses after transfection for 48 h and 72 h [Fig. 5A, D]. miR-17-5p overexpression also significantly decreased the number of migrating and invasive MDA-MB-231 and BT549 cells [Fig. 6A-H], and inhibited wound healing [Fig. 6I-L], indicating that miR-17-5p may function as a tumour suppressor in TNBC.

### ETV1 is involved in miR-17-5p-induced anti-proliferative and anti-migratory effects on TNBC cells

To verify that the direct interaction between miR-17-5p and ETV1 changes the functional phenotype of TNBC cells, we treated MDA-MB-231 and BT549 cells as described above. The cells were then used for proliferation, migration and invasion assays in vitro. As predicted, enhancement of miR-17-5p significantly suppressed the proliferative and migratory capacities of the treated TNBC cells compared to control cells. However, restoration of ETV1 expression in the presence of miR-17-5p significantly recovered the proliferative and migratory capacities of the TNBC cells [Fig. 5 and Fig. 6].

**Fig. 7:**
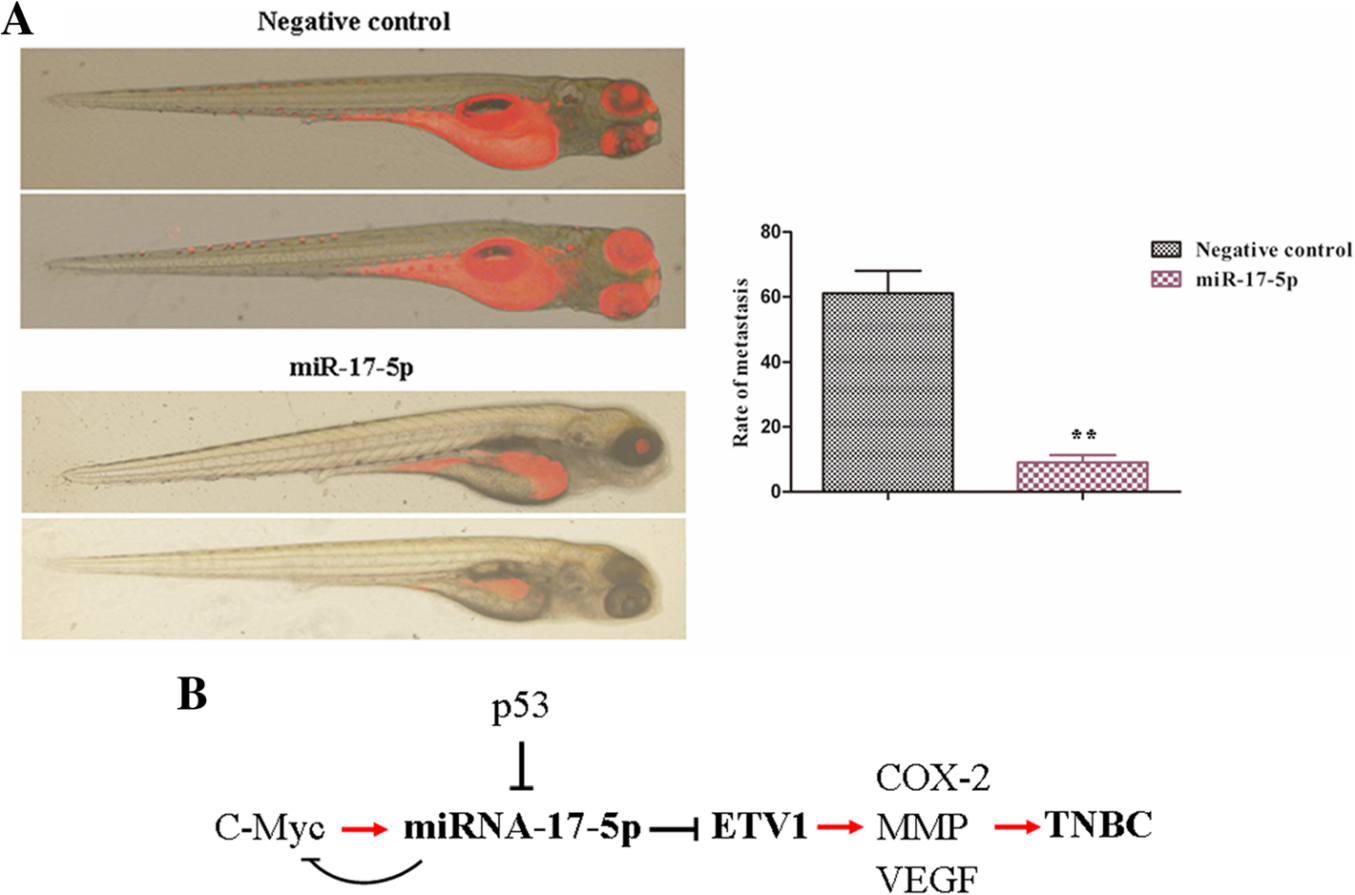
miR-17-5p suppressed TNBC cells metastasis in vivo by targeting ETV1 and the potential pathway. **a**, Control oligos (50 nmol/L) or miR-17-5p mimic (50 nmol/L) transfected MDA-MB-231 cells were labelled with fluorescent dye CM-Dil and then microinjected into the perivitelline spaces of 48-hpf zebrafish embryos. The zebrafish embryos were anaesthetized and imaged using microscopy at 48 h after injection. **b**, miR-17-5p transcription might be activated by c-Myc, and repressed by p53. Up-regulation of miR-17-5p could inhibit ETV1expression, and negative regulate c-Myc transcription. Finally, miR-17-5p could suppress TNBC development by targeting ETV1 through decreasing MMP, COX-2 and VEGF expression. ** *P* < 0.01, compared with the control

Previously, we demonstrated that ETV1 was significantly increased in TNBC cells and could facilitate TNBC cell growth, invasion and migration [6]. To further observe the potential functional link between miR-17-5p and ETV1 in vivo, we utilized a metastatic zebrafish model. MDA-MB-231 cells were transfected with miR-17-5p mimic (50 nmol/L) or negative control oligos (50 nmol/L). Then, the cells were labelled with fluorescent dye CM-Dil and microinjected into the perivitelline spaces of 48-hpf zebrafish embryos. After 2 days, metastases were detected in 61 of 65 embryos injected with control cells, whereas metastases were only observed in 9 of 64 embryos injected with MDA-MB-231 cells overexpressing miR-17-5p [Fig. 7A].

These results demonstrate that ETV1 is involved in the miR-17-5p-mediated negative regulation of TNBC cell proliferation, migration and invasion. Furthermore, up-regulation of miR-17-5p expression can effectively suppress TNBC tumourigenesis by degrading ETV1.

## Discussion

Tumourigenesis is associated with a network of abnormal genes expression. The genes expression patterns and their role are substantially different with specific tumour types. Confirming each factor in the network may promote precise medical treatment. To extend our previous study, we focused on the regulatory miRNAs of ETV1 in TNBC. In this study, we found that ETV1 was a direct target of miR-17-5p. Given the observation that miR-17-5p was down-regulated in TNBC cell lines and clinical tumour tissues in contrast to ETV1, we speculated that up-regulation of miR-17-5p would suppress triple-negative breast tumour cell oncogenic activity by targeting ETV1 and dysregulation of miR-17-5p would be associated with the prognosis of TNBC patients.

miR-17-5p has been linked to tumourigenesis in a broad range of cancers, including hepatocellular carcinoma [19], gastric cancer [20], ovarian cancer [21], prostate cancer [22], and breast cancer [23, 24]. The abundance and function of miR-17-5p vary with tumour type, even if observed in same tumour type. Overexpression of miR-17-5p inhibits hormone-dependent breast cancer cell proliferation by targeting AIB1 or cyclin D1 [16, 25]. A systematic analysis showed that down-regulation of miR-17-5p may be a prerequisite for the onset of TNBC metastasis mediated by TGFβ [26]. However, conflicting data have been reported [27, 28]. We suppose that the partial reason may be tumour heterogeneity. In this study, we showed that miR-17-5p expression levels were significantly down-regulated in TNBC cell lines and clinical tumour tissues. We hypothesize that miR-17-5p decrease in TNBC might be associated with c-Myc or p53 abnormal activity [29, 30]. We observed that enhancement of miR-17-5p in TNBC cells significantly inhibited cell proliferation, migration and invasion. Lower miR-17-5p expression was related to a shorter survival time of TNBC patient. miR-17-5p is a independent favourable prognostic factor for TNBC patient. The results support that miR-17-5p may function as a tumour suppressor in TNBC, which is consistent with the literature [26].

ETV1 contributes to tumourigenic activity through activation of MMP, COX-2 and VEGF expression [3, 4, 31, 32]. Studies in our group demonstrated that ETV1 acts as an oncogene in TNBC, and may be an independent, poor prognostic predictor of the patients [6]. Highly expressed miR-17 enhances melanoma cell motility and migration by repressing translation of the ETV1 protein, which may support the development of metastasis [9]. However, the miR-17 level is significantly lower in GIST. Up-regulation of miR-17 in GIST cell lines inhibits cell proliferation by degrading ETV1 mRNA, which may suppress the tumour progression [12]. These results suggest that one miRNA may function as an oncogene or tumour suppressor gene in a cell type- and context-dependent manner, even if it targets same gene [11]. In the present study, an inverse expression pattern of miR-17-5p and ETV1 in TNBC cell lines and tumour tissues was detected. To reinforce the expression of miR-17-5p in TNBC cell lines not only significantly reduced the expression of ETV1 but also inhibited cell proliferation, migration and invasion, which might suppress the development of TNBC. Moreover, rescue of ETV1 expression in the presence of miR-17-5p significantly restore the cell phenotype. These results indicate that miR-17-5p may influence the behavior of TNBC by regulating ETV1 expression. This was further supported by an in vivo trial using a well-established zebrafish metastatic model. Our results suggest that there might exist a signaling pathway, c-Myc/p53—miR-17-5p—ETV1—MMP, COX-2 and VEGF [Fig. 7B], playing a role in TNBC development [3, 6, 29]. In addition, overexpression of COP1 can significantly reduce the level of ETV1, and turnover TNBC cell phenotype [6, 33]. The mutual phenomenon is the relatively low abundance of COP1 and miR-17-5p in TNBC, which might be the source of TNBC occurrence. Therefore, whether the joint overexpression of both miR-17-5p and COP1 or other suppressor genes is a new strategy for TNBC therapy by targeting ETV1 needs further research. In addition to miR-17-5p, other members of the miR-17-92 cluster, such as miR-19a/b, etc., which are also dysregulated in TNBC, have potential binding sites in ETV1. Future study may clarify whether these miRNAs are involved in modifying TNBC characteristics by targeting ETV1. Recently, cancer stem cells (CSCs) have been suggested as a cause of metastasis and recurrence in breast cancer [34]. To confirm the same modulatory mode between miR-17-5p and ETV1 existing in CSC from TNBC cells would be essential for achieving long term therapeutic success for TNBC patients by targeting these rare cells.

## Conclusion

The current study showed that miR-17-5p functions as a tumour suppressor by targeting ETV1 in TNBC progression and metastasis and is a independent favourable predictor for TNBC patients’ prognoses. These results shed light on TNBC targeted therapy by balancing tumour suppressor genes and oncogenes. However, additional evidences are required to validate the detailed mechanisms of miR-17-5p in TNBC tumourigenesis and as a potential therapeutic target. Furthermore, it would be more convincing if the association between miR-17-5p and ETV1 expression could be confirmed in TNBC clinical samples with a larger cohort.

## Additional files

Additional file 1: Table S1. Primers for coding sequences of ETV1 and miR-17-5p. (DOC 25 kb)

Additional file 2: Table S2. qRT-PCR primer sequences used in this study. (DOC 26 kb)

Additional file 3: Figure S1. Immunohistochemical staining of ETV1 in two representative TNBC tissues. **a, c,** Negative control of ETV1. **b, d,** Positive expression of ETV1. Among the 105 cases of TNBC, 79 cases were ETV1-positive and 26 were ETV1-negative according to the immunoreactivity score described in the text. Scale bar, 10 μm. (TIFF 22543 kb)

Additional file 4: Table S3. Correlation between ETV1 expression and clinicopathologic features of TNBC patients. (DOC 54 kb)

## Abbreviations

CCK-8: Cell Counting Kit-8
IHC: Immunohistochemistry
ISH: In situ hybridization
miRNA: microRNA
OS: Overall survival
pTNM: Pathologic tumor-node-metastasis
qRT-PCR: Quantitative real-time PCR
TNBC: Triple-negative breast cancer
