# Supplementary material for: miR-17-5p suppresses cell proliferation and invasion by targeting ETV1 in triple-negative breast cancer
Source: BMC Cancer. 2017 Nov 10;17:745. doi: 10.1186/s12885-017-3674-x (PMC5681773; doi:10.1186/s12885-017-3674-x)
Supplement: Supplementary file 1 — Primers for coding sequences of ETV1 and miR-17-5p. (DOC 25 kb) [file 12885_2017_3674_MOESM1_ESM.doc]

**Table S1** **Primers for coding sequences of ETV1 and miR-17-5p**

| **Gene name** | Forward (5’-3’) | Reverse (5’-3’) |
| --- | --- | --- |
| **ETV1** | TCCGCTCGAGATGGATGGATTTTATGACC | ATCGGGATCCACATACACGTAGCCTTCGTTG |
| **miR-17-5p** | GCTGAATTTGTATGGTTTATAGTTGTTA | GCACCTTAGAACAAAAAGCACT |
