# Supplementary material for: miR-17-5p suppresses cell proliferation and invasion by targeting ETV1 in triple-negative breast cancer
Source: BMC Cancer. 2017 Nov 10;17:745. doi: 10.1186/s12885-017-3674-x (PMC5681773; doi:10.1186/s12885-017-3674-x)
Supplement: Supplementary file 2 — qRT-PCR primer sequences used in this study. (DOC 26 kb) [file 12885_2017_3674_MOESM2_ESM.doc]

**Table S2** **qRT-PCR primer sequences used in this study**

| **Gene name** | Forward (5’-3’) | Reverse (5’-3’) |
| --- | --- | --- |
| **ETV1** | CGCAGTCCATACCAGATAGCAGC | TGGCATCGTCGGCAAAGGAG |
| **GAPDH** | GAAGGTGAAGGTCGGAGTC | GAAGATGGTGATGGGATTTC |
| **miR-17-5p** | CTCTTACAGTGCAGGTAGAAAA | Universal primer from TAKARA |
| **U6** | ACGCAAATTCGTGAAGCGTT | Universal primer from TAKARA |
