# Supplementary figures and images for: miR-17-5p suppresses cell proliferation and invasion by targeting ETV1 in triple-negative breast cancer
Source: BMC Cancer. 2017 Nov 10;17:745. doi: 10.1186/s12885-017-3674-x (PMC5681773; doi:10.1186/s12885-017-3674-x)

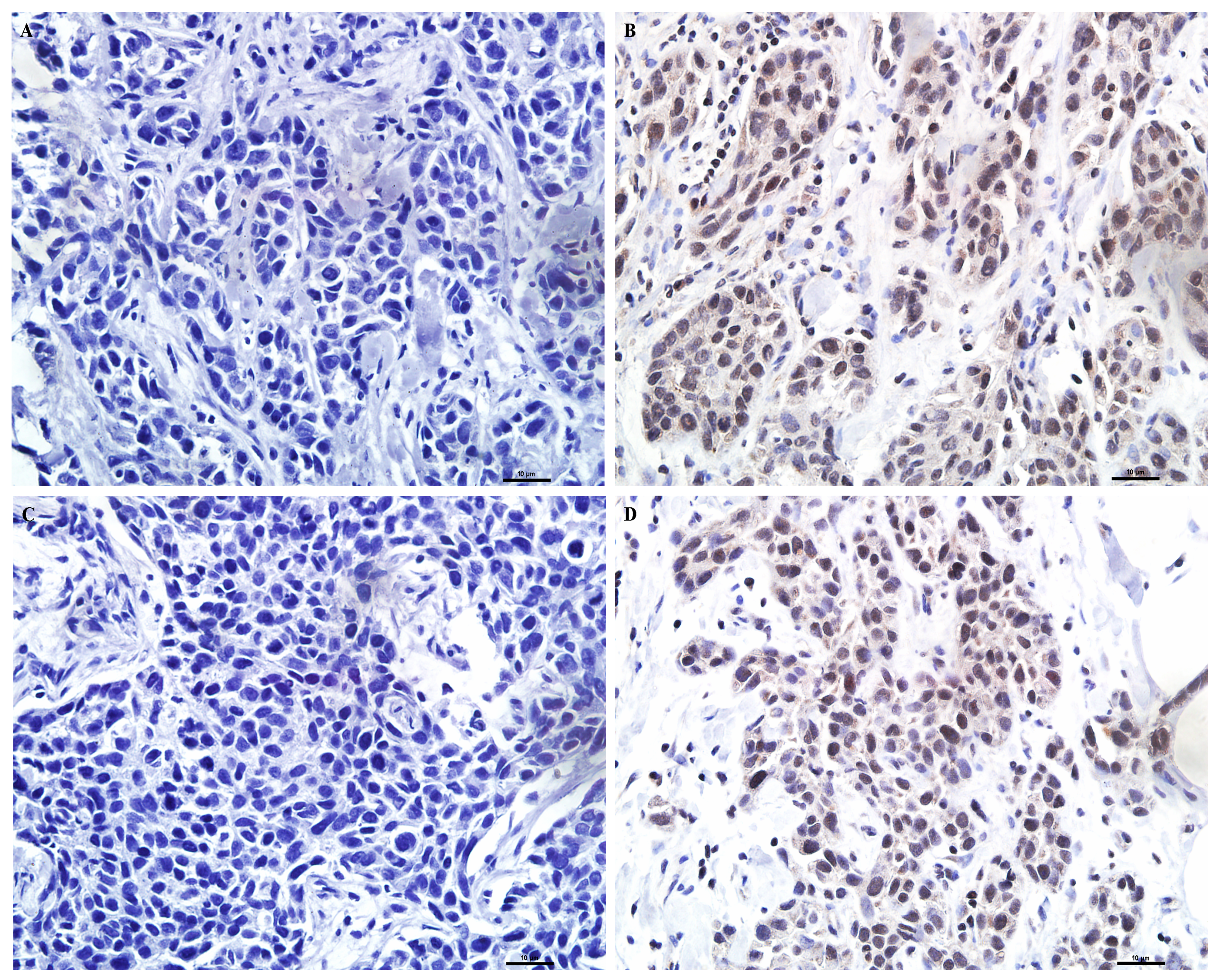

Supplement: Supplementary file 3 — Immunohistochemical staining of ETV1 in two representative TNBC tissues. a, c, Negative control of ETV1. b, d, Positive expression of ETV1. Among the 105 cases of TNBC, 79 cases were ETV1-positive and 26 were ETV1-negative according to the immunoreactivity score described in the text. Scale bar, 10 μm. (TIFF 22543 kb) [file 12885_2017_3674_MOESM3_ESM.tif]
