# Supplementary material for: miR-17-5p suppresses cell proliferation and invasion by targeting ETV1 in triple-negative breast cancer
Source: BMC Cancer. 2017 Nov 10;17:745. doi: 10.1186/s12885-017-3674-x (PMC5681773; doi:10.1186/s12885-017-3674-x)
Supplement: Supplementary file 4 — Correlation between ETV1 expression and clinicopathologic features of TNBC patients. (DOC 54 kb) [file 12885_2017_3674_MOESM4_ESM.doc]

**Table S3** **Correlation between ETV1 expression and clinicopathologic features of TNBC patients**

| **Characteristics** | **n** | **ETV1** | | ***P* value** |
| --- | --- | --- | --- | --- |
|  |  | **Negative** | **Positive** |  |
| **Age (y)** |  |  |  |  |
| ≤ 50 | 63 | 14 (22%) (22%) | 49 (78%) | 0.460 |
| > 50 | 42 | 12 (29%) | 30 (71%) |  |
| **Tumor Si**z**e** |  |  |  |  |
| ≤ 2cm | 19 | 5 (26%) | 14 (74%) | 0.535 |
| > 2cm | 86 | 21 (24%) | 65 (76%) |  |
| **Pathological**  **type** |  |  |  |  |
| Invasive ductal carcinomas | 93 | 21 (23%) | 72 (77%) | 0.139 |
| Others | 12 | 5 (42%) | 7 (58%) |  |
| **TNM Stage** |  |  |  |  |
| I | 6 | 3 (50%) | 3 (50%) | 0.045^*^ |
| II | 34 | 12 (35%) | 22 (65%) |  |
| III | 65 | 11 (17%) | 54 (83%) |  |
| **Lymph node metastasis** |  |  |  |  |
| 0-4 | 45 | 17 (38%) | 28 (62%) | 0.028^*^ |
| 5-8 | 39 | 6 (15%) | 33 (85%) |  |
| ≥ 9 | 21 | 3 (14%) | 18 (86%) |  |
| **Relapse** |  |  |  |  |
| No | 67 | 23 (34%) | 44 (66%) | 0.002^*^ |
| Yes | 38 | 3 (8%) | 35 (92%) |  |

*means *P* < 0.05; Fisher exact test.
